# Anti-proliferative effect of extremely low frequency electromagnetic field on preneoplastic lesions formation in the rat liver

**DOI:** 10.1186/1471-2407-10-159

**Published:** 2010-04-24

**Authors:** Mónica Noemí Jiménez-García, Jaime Arellanes-Robledo, Diana Ivette Aparicio-Bautista, Miguel Ángel Rodríguez-Segura, Saúl Villa-Treviño, Juan José Godina-Nava

**Affiliations:** 1 Department of Physics Center of Research and Advanced Studies of the National Polytechnic Institute, Mexico City, Mexico; 2 Department of Cell Biology, Center of Research and Advanced Studies of the National Polytechnic Institute, Mexico City, Mexico

## Abstract

**Background:**

Recently, extremely low frequency electromagnetic fields (ELF-EMF) have been studied with great interest due to their possible effects on human health. In this study, we evaluated the effect of 4.5 mT - 120 Hz ELF-EMF on the development of preneoplastic lesions in experimental hepatocarcinogenesis.

**Methods:**

Male Fischer-344 rats were subjected to the modified resistant hepatocyte model and were exposed to 4.5 mT - 120 Hz ELF-EMF. The effects of the ELF-EMF on hepatocarcinogenesis, apoptosis, proliferation and cell cycle progression were evaluated by histochemical, TUNEL assay, caspase 3 levels, immunohistochemical and western blot analyses.

**Results:**

The application of the ELF-EMF resulted in a decrease of more than 50% of the number and the area of γ-glutamyl transpeptidase-positive preneoplastic lesions (*P *= 0.01 and *P *= 0.03, respectively) and glutathione S-transferase placental expression (*P *= 0.01). The number of TUNEL-positive cells and the cleaved caspase 3 levels were unaffected; however, the proliferating cell nuclear antigen, Ki-67, and cyclin D1 expression decreased significantly (*P *≤ 0.03), as compared to the sham-exposure group.

**Conclusion:**

The application of 4.5 mT - 120 Hz ELF-EMF inhibits preneoplastic lesions chemically induced in the rat liver through the reduction of cell proliferation, without altering the apoptosis process.

## Background

Electromagnetic fields have been employed as useful tools in medical diagnosis. Recently, the use of electromagnetic fields has been expanded to therapeutic purposes because their interactions with living matter produce effects that initiate, accelerate or inhibit biological processes. Frequencies below 300 Hz are known as extremely low frequency electromagnetic fields (ELF-EMF) and do not have enough energy to break molecular bonds; for example, they do not cause direct damage to DNA [1]. Additionally, ELF-EMF are non-invasive and non-ionizing and even have non-thermal effects on cells and tissues. These properties have led to studies of the influence of ELF-EMF on the development of various diseases, including cancer.

While some researchers associate ELF-EMF exposure with carcinogenesis [2,3], other studies of experimental models and human cancers have shown that ELF-EMF do not increase the risk of several cancer types, including liver cancer, and that treatment with tumor-specific frequencies is feasible and well tolerated and may have biological efficacy in patients with advanced tumors [4-6]. Moreover, the exposure of female C3H/HeJ mice bearing mammary adenocarcinoma to a frequency of 120 Hz at intensities of 4 and 5 mT resulted in a significant reduction in the growth of the tumors, which is a phenomenon associated with angiogenesis inhibition [7]. The exposure of female athymic nude mice with human breast cancer xenografts to a frequency of 120 Hz with an intensity of 15 mT, either alone or in combination with gamma radiation, resulted in decreased growth and reduced vascularization of the tumors [8]. Similarly, the effect of 50 Hz at 0.5 μT and 0.5 mT on the development of chemically induced foci in rat livers showed a slight inhibition of their formation [9]. This evidence that ELF-EMF inhibit carcinogenesis is not convincing, and the exact molecular mechanisms that account for its effects must be validated. The purpose of this study was to use the modified resistant hepatocyte model (MRHM), which induces a rapid proliferation of altered hepatocytes to form preneoplastic lesions in the rat liver [10], as a reliable model to seek information concerning to the effects of ELF-EMF on hepatocarcinogenesis. We hypothesized that the development of preneoplastic lesions chemically induced in rat livers could be affected by 4.5 mT - 120 Hz ELF-EMF exposure. Our results showed that this exposure clearly inhibits the development of preneoplastic lesions through the reduction of cell proliferation, a characteristic alteration that has been found previously in the induction of experimental hepatocarcinogenesis [11,12]. Thus, our finding could be the basis for the design of strategies and clinical applications of ELF-EMF for the treatment of hepatocellular carcinoma.

## Methods

### Reagents and antibodies

N-Diethylnitrosamine (DEN), 2-acetylaminofluorene (2AAF), γ-glutamyl-4-methoxy-2-naphthylamine (GMNA), glycyl-glycine and 4-benzoylamino-2,5-diethoxybenzene-diazonium chloridae hemi [zinc chloride] salt (Fast Blue BB salt) were obtained from SIGMA (St. Louis, MO, USA). A DeadEnd™ Colorimetric TUNEL System kit was purchased from Promega (Madison, WI, USA), and a complete proteases inhibitor cocktail was acquired from Roche (Indianapolis, IN, USA). Anti-glutathione S-transferase placental (GST-p) and Universal LSAB™ plus kit were obtained from DAKO (Carpinteria, CA, USA). Anti-PCNA and DAB Plus Substrate kit were purchased from Zymed (San Francisco, CA, USA). Anti-cyclin D1 was obtained from Bio SB (Santa Barbara, CA, USA), anti-Ki-67 was acquired from Cell Marque Corp (Austin, TX, USA), anti-caspase 3 was purchased from Cell Signaling (Danvers, MA, USA), and anti-actin was obtained from CINVESTAV, Mexico City.

### Experimental design

**Figure 1:**
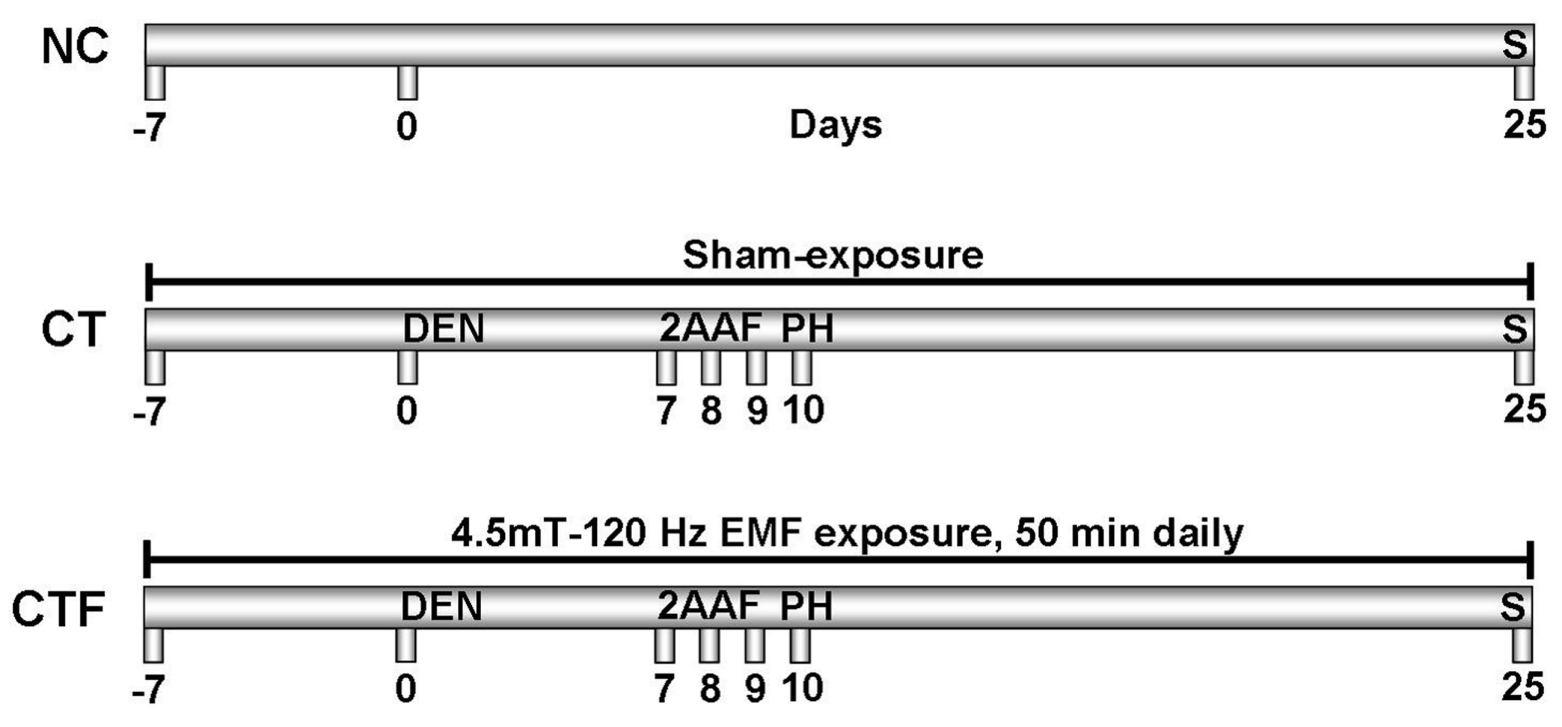
**Schematic representation of the carcinogenic treatment and ELF-EMF exposure**. The sham-exposure or exposure to ELF-EMF was applied before and during carcinogenic treatment, as indicated in schemes of CT and CTF groups, respectively. NC, normal control; CT, carcinogenic treatment and sham-exposure; CTF, carcinogenic treatment plus ELF-EMF exposure. S, sacrifice; DEN, N-diethylnitrosamine; 2AAF, 2-acetylaminofluorene; PH, partial hepatectomy; *n *= 6 for each group.

Male Fischer-344 rats weighing 160 to 200 g, obtained from the Production Unit of Experimental Laboratory Animals (UPEAL-CINVESTAV, México D.F., Mexico), were fed *ad libitum *and housed in a controlled environment (12 h light/12 h dark cycle; room temperature was maintained at 22 ± 2°C, with relative humidity at 55 ± 10%). The experiments were performed in accordance with the guidelines of the Institutional Animal Care and Use Committee of the Official Mexican Standard NOM-062-ZOO-1999. For carcinogenic treatment, MRHM was used [11,13]. The rats were initiated with a necrogenic dose of DEN (200 mg/kg of body weight, i.p.), and 7 days later, 2AAF was orally administered at 20 mg/kg per dose for 3 consecutive days before a partial hepatectomy (PH) as shown Figure 1. Three groups of 6 rats each one, were randomized. The first group, which was used as the normal control did not receive treatment, NC group. The second group, which was used as the positive control for carcinogenesis, was subjected to the carcinogenic treatment and was kept under the same stress conditions without electromagnetic field exposure (sham-exposure) throughout the experiment; in this case the equipment was turned off, CT group. The third group, additionally to the carcinogenic treatment, was exposed to ELF-EMF according to "Animal exposure" section, CTF group. For this group, the administrations of DEN, 2AAF and PH were carried out before ELF-EMF exposure schedule. The animals were sacrificed on day 25 after carcinogenesis initiation (Figure 1). All of the rats from each group were used for all determinations. Biochemical and molecular evaluations were performed under blind conditions by expert people not involved in the animal exposure.

### System of ELF-EMF exposure

**Figure 2:**
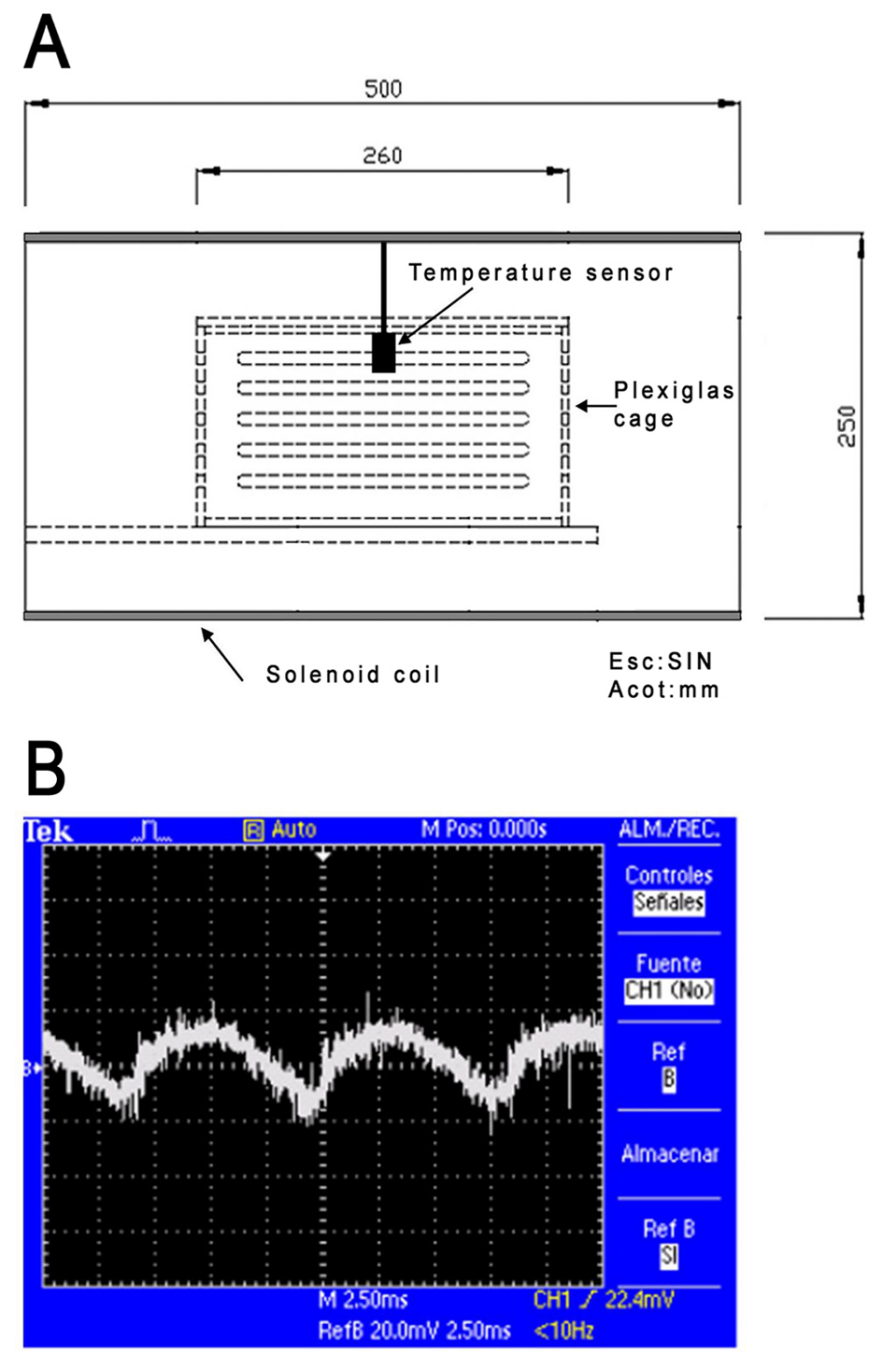
**Scheme of the temperature sensor location and waveform signal**. (**A**) The temperature sensor was placed in the center and inside of the cage where animals were sham-exposed or exposed to ELF-EMF. (**B**) Shows the waveform recorded by the oscilloscope. The signal was measured in the effective volume where animals were exposed.

A uniform and homogeneous ELF-EMF of 120 Hz was generated by a solenoid coil with 1900 turns in two layers and with AWG 20-gauge copper-enameled magnetic wire in a diameter of 25 cm, which was driven by an alternating current source (Staco Variac variable transformer 511, ISE Inc; Cleveland, OH, USA). The equipment was controlled by a computer. The coil generated 0.1 - 4.5 mT in its center, where the rats received the exposure. The magnetic flux density was measured using a Gauss/Teslameter (Hall effect gaussmeter F.W. BELL 5070, SYPRIS Test & Mesurements; Orlando, FL, USA); the signal parameters were monitored by an inductive coil that was connected in parallel to a resistance by an oscilloscope (Tektronix TDS2024, TEKTRONIX Inc; Beaverton, OR, USA). The current flow was measured by an alternating current meter that was connected in series with the solenoid (Digital multimeter Tech TM-178, Techman Electronics Inc; La Verne, CA, USA). When the animals were exposed or had sham-exposure, the temperature inside the solenoid was monitored by a temperature sensor (LM35 IC: Integrated Circuit, National Semiconductor Corporation Americas; Santa Clara, CA, USA). The temperature sensor location and the shape of signal wave are shown in Figure 2.

### Animal exposure

During the exposure, CTF group was divided in two subgroups of three rats each one and placed in a Plexiglas cage (16 × 16 × 25 cm) inside of solenoid. Each subgroup was exposed to 4.5 mT - 120 Hz ELF-EMF for 50 min daily as shown Figure 1; the first group was exposed from 10:00 - 10:50 h and the second was from 12:00 - 12:50 h, alternating them on this schedule, during 32 consecutive days (from 7 days before carcinogenic treatment until 25 days after). After exposure all animals were returned to their home cages. We decided to apply the ELF-EMF from 7 days before starting the carcinogenic treatment, because our initial approach was to assess its effect on the inhibition of preneoplastic lesions development in rat liver, as a strategy to prevent the disease in the early stages of its development.

### GGT histochemical staining

Histological liver sections of 15 μm were obtained in a cryostat (Slee Cryostat MTC, Germany). For each animal 3 tissue sections were analyzed randomly. The preneoplastic lesions in the rat livers were observed by the detection of γ-glutamil transpeptidase (GGT) activity as previously described [14]. Briefly, sections were fixed in absolute ethanol for 10 min at -20°C; the fixation was followed by the addition of GMNA, glycyl-glycine and Fast Blue BB salt in a 100 mM Tris base for 30 min at room temperature. Subsequently, the staining was fixed with cupric sulfate for 2 min. Finally, images of the liver tissue were captured with a digital camera in a microscope, and the GGT-positive lesions were quantified by image analysis software (analySIS Soft Imaging System GmbH). The experimental procedure was carried out at the same time for all comparative groups.

### TUNEL assay and immunohistochemical analysis

Liver tissue sections, 4 μm thick, were deparaffinized and hydrated gradually. DNA fragmentation was determined by a Colorimetric TUNEL System kit, according to the manufacturer's instructions and a tissue treated with DNase I was used as a positive control. For immunohistochemical analyses 4 sequential sections per rat were analyzed; antigens were unmasked by immersing the sections in 0.1 M sodium citrate buffer (pH 6) in a heated water bath for 15 min. Then, endogenous peroxidase activity was blocked with 0.3% H_2_O_2 _in methanol. Primary antibodies anti-GST-p, anti-PCNA, anti-Ki-67 and anti-cyclin D1 were incubated overnight at 4°C. After a standard staining protocol using Universal LSAB™ plus kit and a DAB Plus Substrate kit as the chromogen, the sections were lightly counterstained with hematoxylin, dehydrated and mounted. As a positive control for PCNA, Ki-67 and cyclin D1, liver sections from one rat subjected to partial hepatectomy and sacrificed after 24 h, were used to observe high proliferation levels. Tissues images were captured by optical microscopy (Olympus 1X70, Olympus Europa GmbH, Hamburg, Germany). Then, positive cells for PCNA, Ki-67 and cyclin D1 were quantified in ten randomly selected fields (magnification 10×) per individual sample, and the numbers of positive cells/mm^2 ^were calculated using image analysis software (analySIS Soft Imaging System GmbH). For detection of each protein, the immunostaining protocol was carried out at the same time for all comparative groups.

### Western blot analysis

For proteins extraction, tissues were homogenized with lysis buffer (10 mM Tris-Cl pH 7.4, 150 mM NaCl, 1% Triton-X100 and proteases inhibitor cocktail), the cell lysates were centrifuged at 15,000 *g *for 30 min, and the supernatant was stored at -80°C. The procedure was performed at 4°C. Proteins were separated by SDS-PAGE and were transferred to a PVDF membrane. The protein of interest was visualized using the indicated antibody and a chemiluminescence system. Anti-actin was used as a protein loading control.

### Statistical analysis

Statistical differences were obtained between the carcinogenic treatment and the carcinogenic treatment plus electromagnetic field exposure using the Student's *t*-test. Data were expressed as the mean ± the standard error of the mean (SEM). Differences were considered significant when *P *< 0.05.

## Results

### General observations

The average body and relative liver weights of rats treated either with CT or CTF were not different throughout the experiment (data not shown). We also monitored the temperature of sham-exposure (CT) or exposure (CTF) groups during the 32 days and their values were recorded at specific times shown in Table 1. The three degree differential temperature between the groups was due to the solenoid was turned off when the sham-group was exposed.

**Table 1:** Average values of the temperature recorded during the exposure time

	Treatment group	
	CT	CTF
	
Time/min	T/°C	T/°C
0	23.7 ± 0.4	23.7 ± 0.4
25	23.9 ± 0.6	25.8 ± 0.6
35	24.2 ± 0.6	26.7 ± 0.7
45	24.0 ± 0.5	27.1 ± 0.6
50	24.1 ± 0.6	27.0 ± 0.7

All values are expressed as the mean ± SEM

### ELF-EMF exposure inhibits preneoplastic lesions development

**Figure 3:**
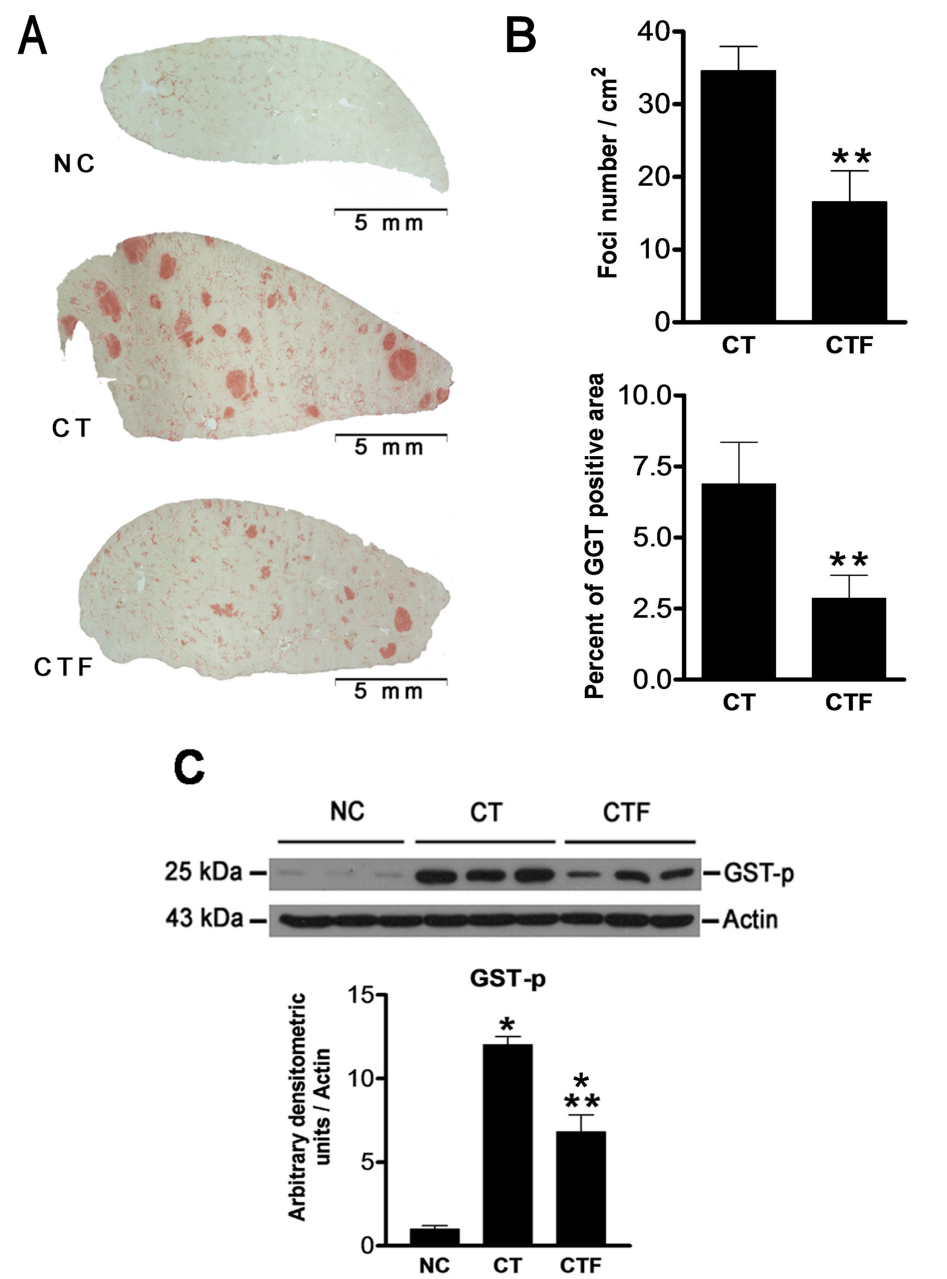
**Effect of ELF-EMF exposure on GGT-positive lesions and GST-p expression**. (**A**) Representative liver sections of the NC group, CT group and CTF group. Scale bars, 5 mm. (**B**) Quantification of the foci number/cm^2 ^and percent of the GGT-positive area. (**C**) Western blot analysis for GST-p expression. GST-p was normalized with actin expression used as the loading control. The expression of NC was adjusted to one in the densitometric units scale. Statistically different from *NC and **CT, *P *< 0.05. Data are expressed as the mean ± SEM; *n *= 6 for each group.

Animals subjected to the CTF protocol showed a significant reduction in both the number and the area of GGT-positive lesions as compared to rats subjected to the CT protocol (Figure 3A). Table 2 shows the average value of foci number per cm^2 ^and the percent of area GGT-positive of each animal subjected to different experimental protocols. The number and area of GGT-positive lesions were reduced by 52.2 and 58.4% (*P *= 0.01 and *P *= 0.03), respectively (Figure 3B). Similarly, western blot analysis (Figure 3C) showed that GST-p expression was reduced by 43.3% (*P *= 0.01). These results indicate that the application of 4.5 mT - 120 Hz ELF-EMF inhibits the development of preneoplastic lesions induced by the hepatocarcinogenesis experimental protocol.

**Table 2:** Effect of ELF-EMF exposure on foci number per cm^2 ^and the percent of area GGT-positive

Group	# animal	**Foci number/cm**^2^	% of GGT-positive area
CT	1	38.71 ± 3.3	6.93 ± 0.4
	2	16.31 ± 2.6	2.27 ± 0.5
	3	39.22 ± 11.4	5.63 ± 1.2
	4	31.42 ± 1.5	12.76 ± 0.8
	5	40.51 ± 9.0	8.77 ± 1.7
	6	28.66 ± 6.2	4.98 ± 1.1
			
CTF	1	0.65 ± 0.6	0.07 ± 0.1
	2	8.13 ± 3.5	1.18 ± 0.5
	3	27.45 ± 5.7	4.59 ± 0.9
	4	14.85 ± 2.6	2.38 ± 0.6
	5	24.40 ± 2.2	3.80 ± 0.2
	6	14.50 ± 2.5	4.52 ± 0.7

Values represent the mean ± SEM of three liver sections evaluated per animal.

### ELF-EMF exposure did not induce apoptosis

**Figure 4:**
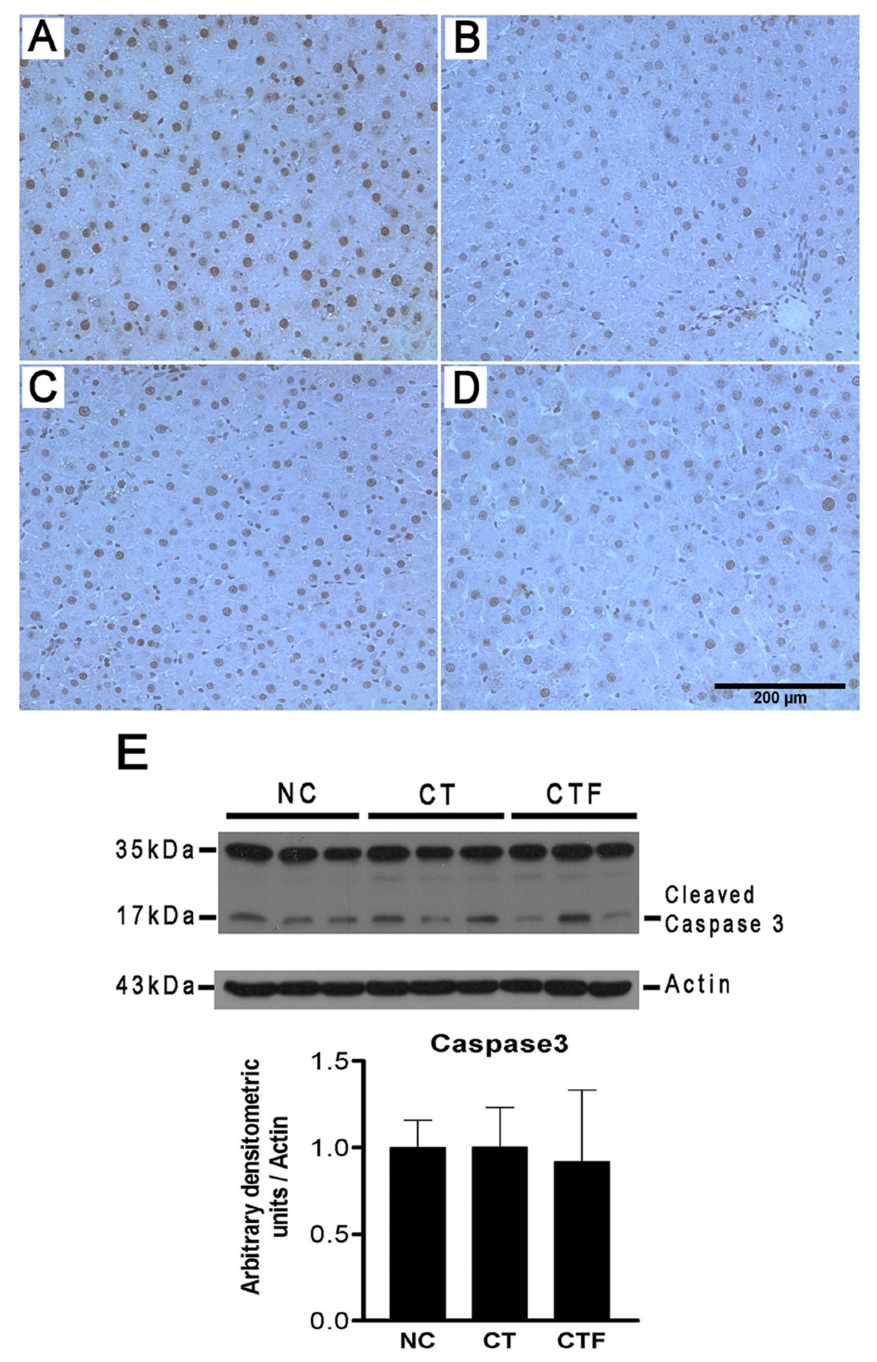
**Effect of ELF-EMF exposure on apoptosis**. Representative liver sections of each treatment are shown. (**A**) Positive control tissue that was treated with DNase I. (**B**) Normal control tissue. (**C**) CT protocol. (**D**) CTF protocol; images magnification 200×. (**E**) Western blot analysis for cleaved caspase 3 levels. Caspase 3 was normalized with actin expression used as the loading control. The expression of NC was adjusted to one in the densitometric units scale; *n *= 6 for each group.

To evaluate the effect of ELF-EMF exposure on the apoptosis induction of altered hepatocytes, we used two different procedures applied to the three groups. First, cells with DNA fragmented were identified in tissues by a TUNEL assay. Representative tissue sections of each treatment are depicted in Figure 4. Although a slight increase of TUNEL-positive cells is observed in the rat tissues of CT group (Figure 4C), these were not different from those of rats in the NC group or those in the CTF group (Figures 4B and 4D). According to this result, the levels of cleaved caspase 3 were not affected by either the CT or the CTF protocols as compared to the NC protocol (Figure 4E). These results indicate that the application of 4.5 mT - 120 Hz ELF-EMF does not induce either DNA fragmentation or the caspase-3 activation, indicating that it does not promote apoptosis of altered hepatocytes.

### The application of 4.5 mT - 120 Hz ELF-EMF inhibits proliferation during in vivo hepatocarcinogenesis

**Figure 5:**
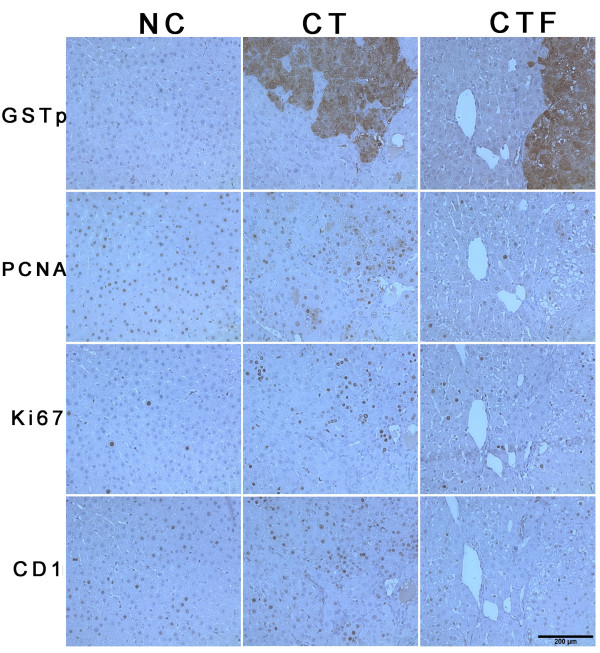
**Immunohistochemical analysis of GST-p, PCNA, Ki-67 and cyclin D1 expression**. Four serial liver sections from each treatment are shown in columns. Immunostaining for each protein is displayed in rows. GST-p detection shows the preneoplastic lesions for localization of PCNA, Ki-67 and cyclin D1 (CD1) expression. NC, normal control; CT, carcinogenic treatment; CTF, carcinogenic treatment plus ELF-EMF exposure; images magnification 200×; *n *= 6 for each group.

**Figure 6:**
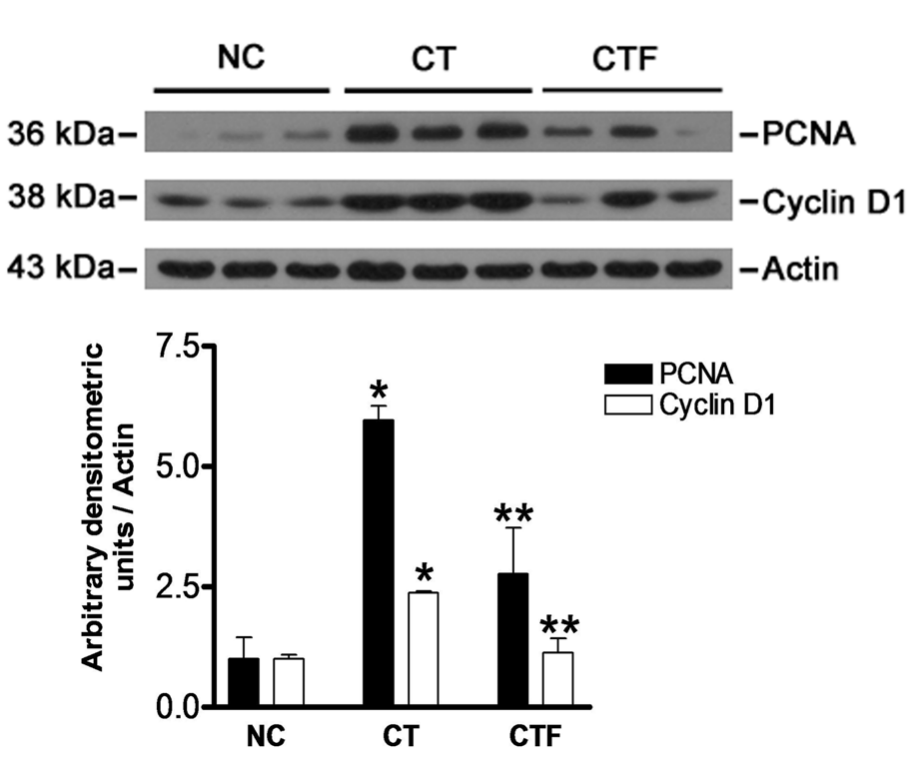
**Effect of ELF-EMF exposure on PCNA and cyclin D1 expression**. Western blot analysis for PCNA and cyclin D1 expressions. Both expressions were normalized with actin expression used as the loading control. The expression of NC was adjusted to one in the densitometric units scale. Statistically different from *NC and **CT, *P *< 0.05. Data are expressed as the mean ± SEM; *n *= 6 for each group.

To determine whether the application of 4.5 mT - 120 Hz ELF-EMF had an effect on cell proliferation, which is a characteristic alteration from the induction of experimental hepatocarcinogenesis, we analyzed the expression of PCNA, which participates in replication and DNA repair, and the expression of Ki-67, a specific replication marker, which participates in all active phases of the cell cycle except for the G0 phase. Moreover, PCNA and Ki-67 are used to determinate the proliferating activity of cancer cells [15-17]. Figure 5 shows an immunohistochemical analysis of sequential sections of the same piece of liver tissue in which the expression of PCNA and Ki-67 proteins in GST-p-positive preneoplastic lesions can be seen. The positive labels of PCNA and Ki-67 were not limited to preneoplastic lesions; they were also detected in whole tissues of the rats subjected to the CT and CTF protocols. We observed, however, a clear reduction in the number of positively labeled cells, which was corroborated through a quantitative analysis, as shown in Table 3. The numbers of PCNA- and Ki-67-positive cells per mm^2 ^were decreased in the CTF protocol as compared to the CT protocol by 87.88% (*P *= 0.03) and 86.97% (*P *= 0.004), respectively. The nucleus labels of hepatocytes situated within the limits of the preneoplastic lesions for both the CT and CTF protocols were more intense. Figure 6 shows a western blot analysis that revealed a similar diminution of PCNA expression (53.62%, *P *= 0.03).

**Table 3:** Number of PCNA-, Ki-67- and cyclin D1-positive cells/mm^2^

	Cell number/mm^2^*		
	
Treatment group	PCNA	Ki-67	cyclin D1
NC	22.36 ± 1.05	7.02 ± 3.24	49.31 ± 2.07
CT	492.03 ± 76.36^**a**^	192.74 ± 15.49^**a**^	265.39 ± 23.95^**a**^
CTF	59.59 ± 10.38^**b**^	25.11 ± 3.32^**b**^	44.75 ± 5.36^**b**^

*Mean ± SEM from ten fields per rat tissue, captured at 100× magnification, *n *= 3

^**a**^Statistically different from the NC group, *P *< 0.05

^**b**^Statistically different from the CT group, *P *< 0.05

In order to determine the underlying mechanism of the proliferation inhibition mediated by the application of 4.5 mT - 120 Hz ELF-EMF, we investigated the effect of this application on cyclin D1 expression, a cell cycle regulator protein responsible for the transition from G1- to S-phase in both normal regulation of the cell cycle and development of human cancers [18]. Thus, the coexistence of GST-p and cyclin D1 in serial sections of tissue was analyzed in a similar manner to that of the PCNA and Ki-67 expressions. As shown in Figure 5 and Table 3, the number of cyclin-D1-positive cells per mm^2 ^was decreased for the CTF protocol as compared to the CT protocol by 83.13% (*P *= 0.008). Similar to PCNA and Ki-67, cyclin D1-positive cells were observed in whole tissues of rats that were subjected to both the CT and CTF protocols. Figure 6 shows a western blot analysis that confirmed the decrease of the expression of cyclin D1 (53.43%, *P *= 0.01). Together, these results indicate that the inhibition of preneoplastic lesions development induced by the application of 4.5 mT - 120 Hz ELF-EMF in rat livers was associated with a reduction of cell proliferation and suggest that ELF-EMF protects hepatocytes from the increased proliferation induced by the carcinogenic treatment.

## Discussion

Cancer cells manifest at least six main physiological alterations, which are governed by the deregulation of hundreds of genes that play a role in tumor initiation and progression and that collectively dictate malignant growth. One of the physiological alterations of cancer cells is their continuous growth; events associated with the increase in cell proliferation are the loss of apoptotic mechanisms and cell cycle continuity [19]. We have observed that, in the early hepatocarcinogenesis induced by the MRHM, cell proliferation is highly increased, which affects molecules that participate in cell cycle continuity and results in the formation of preneoplastic lesions that can progress toward hepatocellular carcinoma [11,12,20]. In this study, rats subjected to MRHM were exposed to 4.5 mT - 120 Hz ELF-EMF in order to evaluate the effects of the electromagnetic fields on the development of liver preneoplastic lesions. Our results showed that the 4.5 mT - 120 Hz ELF-EMF exposure significantly reduced both the number and the area of GGT-positive lesions and GST-p expression, which are two of the best markers for identifying preneoplastic lesions. We have validated this phenomenon, which has been observed previously, although only as a slight inhibition of the liver foci [9]. Although both studies involved hepatocarcinogenesis protocols, there are several differences in the waveform of their stimulus as compared to ours, such as the time of ELF-EMF exposure. Another important difference is that, in our model, the hepatocarcinogenesis treatment employed DEN, 2-AAF and PH without any additional intervention after PH, whereas, in the protocol used by Rannug and coworkers, phenobarbital, a promoter of cell growth, was administered continuously throughout the 12 weeks of their experiment in addition to DEN and PH. It could be the case that the continuous promotion of altered cells in preneoplastic nodules counteracts the effects of the ELF-EMF, which resulted in a slight inhibition of the liver foci. In this work we clearly validated that ELF-EMF inhibits significantly the development of preneoplastic lesions. Even though the effects were significant, we observed a temperature differential between comparatives groups due to the solenoid was turned off when the sham-group was exposed; thus, we can not discard that this variable could has or not effect on the results. New experiments are required to determine this important fact.

Some authors have suggested the therapeutic use of ELF-EMF for cancer treatment because, in different experimental models, ELF-EMF have been able to inhibit the growth of cancer cell lines and tumors; however, few *in vivo *experiments have been performed to investigate the molecular mechanisms of ELF-EMF in cancer development [8,21-24]. The anti-carcinogenic effect of ELF-EMF could result from the inhibition of cell proliferation and/or apoptosis induction. *In vitro *studies have reported the pro-apoptotic action of ELF-EMF and that this effect is associated with an increase in the number of annexin-V- and TUNEL-positive cells and caspase 3 activity [21,25]. Previously, we observed that our hepatocarcinogenesis model does not induce apoptosis of altered hepatocytes evaluated at different points until 25 days after cancer initiation [11,12]. Using this model, we evaluated the pro-apoptotic effect of ELF-EMF exposure through TUNEL assays and cleaved caspase 3 levels, but we did not find any differences in the apoptosis status between the CT and CTF groups. These data indicate that the application of 4.5 mT - 120 Hz ELF-EMF does not induce either DNA fragmentation or the caspase-3 activation, indicating that it does not promote apoptosis of altered hepatocytes.

During induction of liver cancer using the MRHM, the proliferation of hepatocytes increases; therefore, we tested whether 4.5 mT - 120 Hz ELF-EMF exposure affects the expression of PCNA, Ki-67 and cyclin D1. We showed that ELF-EMF exposure decreased the expression of these proteins, suggesting that the ELF-EMF interferes with both the altered cell cycle continuity and DNA synthesis induced by chemical hepatocarcinogenesis. The effects on cyclin D1 expression and other proteins of the cell cycle have also been observed in human diploid amniotic fluid cells, where 1 mT - 50 Hz exposure diminished cyclin D1 expression though a significant cell arrest in the G2/M phase was induced when the electromagnetic field was applied in combination with different doses of ionizing radiation [26].

Since cells in liver preneoplastic lesions have a growth advantage as compared to the surrounding tissue, we also investigated whether PCNA, Ki-67 and cyclin D1 expressions were limited to the area of the lesions that were GST-p positive. We found that these proteins were expressed by cells from the whole tissue, including the preneoplastic lesions and surrounding tissue, although we found a significant reduction in the protein expression of tissues from rats subjected to the CTF protocol as compared to those of the CT protocol. Based on immunohistochemical analysis, the diminutions in the positive cells number for three proteins were more than 80%. This effect was confirmed by western blot analysis for PCNA and cyclin D1 proteins where there was a reduction in the number of positive cells in more than 50%. Together, these results show that 4.5 mT - 120 Hz ELF-EMF exposure affects the development of preneoplastic lesions of the liver and that this effect is associated with the inhibition of the proliferation process.

The correlation between the presence of Ki-67, PCNA and cyclin D1 has already been studied; while PCNA participates in replication and DNA repair and is also closely associated with the cell cycle machinery, Ki-67 is a specific replication marker associated with cell cycle entry given that participates in all active phases of the cell cycle except for the G0 phase [15,16,27]. We evaluated their expression by immunohistochemistry and found that ELF-EMF altered the amounts of positive cells expressing these proteins and we concluded that this effect is associated with the diminishing of cell proliferation. However, given that PCNA takes part else in DNA repair and that the amount of PCNA-positive cells were larger than Ki-67 (see Table 3); if we assume that all Ki-67-positive cells were also PCNA-positive, it is striking to observe that from the subtraction between them ([PCNA - Ki67]), the number of remaining PCNA-positive cells is similar to the number of cyclin D1-positive cells. Thus, the remaining PCNA-positive cells would be involved in DNA repair, but not in proliferation processes. A similar analysis was reported in human myocytes, where was observed that TUNEL-positive cells can simultaneously express PCNA, but not Ki-67 [28]. This also could explain why the slight increase of TUNEL-positive cells observed in CT group, was not confirmed by cleaved caspase-3 detection. Together this information, suggests that the ELF-EMF could be altering the amount of cells bearing DNA damage in the MRHM model. Further studies are required to determine the nature of this possible association.

In our laboratory, we showed that the celecoxib, a non-steroidal anti-inflammatory drug, inhibits the development of preneoplastic lesions through anti-proliferative mechanisms without inducing the apoptosis process [12], similar to the 4.5 mT - 120 Hz ELF-EMF exposure showed in this work. Even though celecoxib is a synthetic molecule and ELF-EMF is an interaction energy, both have anti-carcinogenic effects; however, when celecoxib is administered at high doses increases the risk of cardiovascular events [29]. Given the efficacy shown by ELF-EMF exposure, our results motivate the evaluation of the synergic effect of ELF-EMF in combination with low doses of celecoxib, which could increase their efficacy and minimize the cardiovascular damage [30,31]; this combination of treatments could have an analogous result to the combination of low doses of X-ray or gamma radiation plus ELF-EMF exposure [8,25].

ELF-EMF are able to interact with moving electrons and increase electron transfer rates in chemical reactions [32]. However, the interaction of ELF-EMF exposure with biological systems, from a physical point of view, remains unclear. Nevertheless, a biophysical model has recently been hypothesized. In this model, the action mechanism of the electromagnetic fields in cells occurs through the forced vibration of each of the free ions that exist on both sides of all plasma membranes and that can move across of them using transmembrane proteins, which disrupt the electrochemical balance of the plasma membrane and, therefore, the whole function of the cell [33]. Furthermore, evaluations in experimental models have been established that the electromagnetic fields are able to modulate the intracellular calcium (Ca^2+^) when cellular homeostasis is disrupted [34]. Calcium is a highly versatile intracellular signal that can regulate many different cellular functions, whether normal or pathological; thus, the consequences of Ca^2+ ^signaling depend of steady state between Ca^2+ ^influx, efflux, and storage [35]. Cancer development takes place through rapid proliferation and the continuous increase of altered cells that modify the cellular environment [19], including the flow of ionic charges across the cell membrane, such as Ca^2+ ^flow. Given that several blockers of Ca^2+ ^entry inhibit tumor growth [36], we cannot discount that ELF-EMF could be regulating Ca^2+ ^flow in the cells. Therefore, we can speculate that the interaction of ELF-EMF with ion flows in the membranes of altered cells interferes with processes that are involved in the development of liver preneoplastic lesions, such as the changes in cell cycle continuity induced by DEN, 2AAF and PH. Finally, another possible action mechanism of ELF-EMF could be at radical chemistry levels; in this way we currently are designing experiments to determine this possible effect on electron transfer rates involved in the oxidative stress generated throughout the chemical hepatocarcinogenesis progression and we are also making a quantum-mechanical model, using radical pairs mechanism theory, that could explain this process.

## Conclusion

Our results indicate that the application of 4.5 mT - 120 Hz ELF-EMF affects the early carcinogenesis chemically induced in rat livers, through the reduction of PCNA, Ki-67 and cyclin D1 expressions without inducing apoptosis, which suggests that ELF-EMF regulate cellular homeostasis and inhibit the development of preneoplastic lesions. Finally, considering that hepatocellular carcinoma is a common form of cancer and that its incidence around the world remains high [37], this finding could be the basis for the design of strategies and clinical applications of ELF-EMF to treat this disease, aimed primarily at high-risk populations.

## List of abbreviations

2AAF: 2-acetylaminofluorene; DEN: N-Diethylnitrosamine; ELF-EMF: extremely low frequency electromagnetic fields; GMNA: γ-glutamyl-4-methoxy-2-naphthylamine; GGT: γ-glutamil transpeptidase; MRHM: modified resistant hepatocyte model; PH: partial hepatectomy; SEM: standard error of the mean.

## Competing interests

The authors declare that they have no competing interests.

## Authors' contributions

MNJG designed and performed the study and drafted the manuscript. JAR and DIAB participated in western blotting and immunohistochemical determinations and made substantial contributions to the study design. MARS designed the software and the electronic diagram of the electromagnetic field equipment. SVT and JJGN led and supervised the entire study. All the authors read and approved the final version of the manuscript.

## Pre-publication history

The pre-publication history for this paper can be accessed here:

http://www.biomedcentral.com/1471-2407/10/159/prepub
